# Oncolytic Viruses and Hematological Malignancies: A New Class of Immunotherapy Drugs

**DOI:** 10.3390/curroncol28010019

**Published:** 2020-12-25

**Authors:** Vanessa Innao, Vincenzo Rizzo, Andrea Gaetano Allegra, Caterina Musolino, Alessandro Allegra

**Affiliations:** 1 Department of Human Pathology in Adulthood and Childhood “Gaetano Barresi”, Division of Hematology, University of Messina, 98125 Messina, Italy; vinnao@unime.it (V.I.); andrea.allegra@hotmail.it (A.G.A.); cmusolino@unime.it (C.M.); 2 Department of Clinical and Experimental Medicine, University of Messina, 98125 Messina, Italy; vrizzo@unime.it

**Keywords:** oncolytic virus, hematological disease, immune response, multiple myeloma, acute myeloid leukaemia, lymphoproliferative disorder

## Abstract

The use of viruses for tumour treatment has been imagined more than one hundred years ago, when it was reported that viral diseases were occasionally leading to a decrease in neoplastic lesions. Oncolytic viruses (OVs) seem to have a specific tropism for tumour cells. Previously, it was hypothesised that OVs’ antineoplastic actions were mainly due to their ability to contaminate, proliferate and destroy tumour cells and the immediate destructive effect on cells was believed to be the single mechanism of action of OVs’ action. Instead, it has been established that oncolytic viruses operate via a multiplicity of systems, including mutation of tumour milieu and a composite change of the activity of immune effectors. Oncolytic viruses redesign the tumour environment towards an antitumour milieu. The aim of our work is to evaluate the findings present in the literature about the use of OVs in the cure of haematological neoplastic pathologies such as multiple myeloma, acute and chronic myeloid leukaemia, and lymphoproliferative diseases. Further experimentations are essential to recognize the most efficient virus or treatment combinations for specific haematological diseases, and the combinations able to induce the strongest immune response.

## 1. Introduction

### 1.1. General Considerations on Oncolytic Viruses

The use of viruses for tumour treatment has been imagined in the early years of the last century, when it was reported that virus-related diseases were occasionally leading to a decrease in neoplastic lesions, increasing patients’ survival [1].

Since then, an enormous number of oncolytic viruses (OVs) has been used and several viruses have been examined for tumour treatment [2].

Oncolytic viruses seem to have a specific tropism for tumour cells. In fact, in non-tumour cells viral infection causes the liberation of local interferons (IFNs), generated ensuing the identification of virus components (DNA, RNA, or proteins) by specific intracellular receptors. IFN and correlated cytokines are extensively involved in the stimulation of the acquired immune system and the beginning of programmed cell death, causing inhibition of tumour cells growth. This activation provokes the stimulation of several genes which raise the reduction of the viral load. Viral components also stimulate toll-like receptors (TLR) that themselves provoke antiviral reaction through the stimulation of host cell agents as retinoic acid-inducible gene that influence the rise of IFNs. Moreover, other viral components stimulate the synthesis of other substances such as protein kinase R which hinder protein production and impede viral proliferation. Instead, tumour cells show several genetic modifications that viruses can use for proliferation while an alteration is habitually detected in neoplastic cells’ reaction to IFN [3,4,5].

In the past, these findings were not adequate to start a new type of cancer therapy, due to serious collateral effects and to the absence of knowhows for genetic mutations. In fact, cell selectivity is an intrinsic characteristic of specific viruses, such as reovirus and parvoviruses [6], while for different viruses, such as herpes simplex virus, this characteristic can be genetically obtained [7,8,9].

### 1.2. Families of Oncolytic Viruses

Mathematical models proposed that the perfect OV should have prompt but tumour-specific spread and hold immune escaping tactics [10].

Several families of viruses have been used in the quest for successful vectors or effectors in antitumour treatment. In fact, each of the viruses used has specific traits which can be utilized to enhance oncolytic effectiveness or decrease the side effects of this particular therapeutic approach.

Here are some of the viruses used in the experiments listed below.

There are several features of the adenovirus (Ad) which make it an advantageous viral carrier. Primarily, the genetic material of the virus is constant, and has a great replicating ability. Ads retain a high in vivo transduction effectiveness and collateral effects are very slight with respect to conventional drug treatments and immunotherapies. Although Ad’s receptor is not present in several tumour cells, the fibre/knob domain of the capsid can be altered to readdress Ad connecting to several receptors present on the cell surface [11,12,13]. Moreover, Ad is a nonenveloped virus with a proliferation cycle concluding in destruction of cells of the host organism. This destructive proliferation activity is advantageous for oncolysis [14] with respect to enveloped viruses which conclude proliferation by growing from vital, undamaged host cells.

The influenza virus is a little virus of the Orthomyxoviridae family, known for provoking the influenza [15]. It includes four types, influenza A, B, C, and D viruses [16]. Although the influenza virus can cause durable stimulation of the immune system in humans, it never leads to chronic pathology and mitigated shapes have been reported [17,18].

Myxoma virus (MYXV) is substantially confined to European rabbits, and it is not harmful for other animals, including humans. Notwithstanding its host specificity, it has been demonstrated that MYXV is able to infect and carry out a multiplicity of human tumour cells while saving non cancer cells, including hematopoietic stem cells [19].

The Vesicular Stomatitis Virus (VSV) is an enveloped RNA virus belonging to the Rhabdvovirus family that has been used as a lytic element to treat human tumours. VSV is extremely appealing for virotherapy as its specific tropism allows contamination of a large multiplicity of tumour cells, and its quick proliferation cycle gives a powerful oncolytic strength [20]. Moreover, proliferation of VSV is restricted to tumour cells as these cells generally show alterations in nonspecific immunity components during neoplastic occurrence. In the case of VSV, an inefficient IFN response causing a decreased stimulation of IFN-inducible genes represents the key factor in cell selectivity. Moreover, tumour cells with alterations different signalling pathways such as Ras or Myc or tumour suppressor p53 were observed to be vulnerable to VSV-caused cell destruction [21]. Additionally, tumour-exclusive modifications concerning the cofactor eIF2B have been reported to allow efficient proliferation in tumour cells [22,23,24]. The matrix protein of VSV (VSV-M) is involved in dislocating the cytoskeleton and damages antigen recognition [25]. To elude the strategies implemented by host cells to defend themselves from viral infection, VSV inhibits the synthesis of proteins in several aspects. VSV-M blocks RNA polymerases I–III and inhibits the transfer from the nucleus to the cytoplasm of host cell mRNAs by interface with export proteins such as Rae1 and Nup98 [26,27,28,29,30,31,32]. Furthermore, the action on translation initiation component, eIF4E, prevents the translation of host mRNAs [33].

Reovirus is a RNA virus that is found in the gastrointestinal and upper respiratory tract. Clinically, this virus is not believed to be significant as infections are usually asymptomatic.

Measles virus (MV) has been documented to target several tumour types. Chen et al. reported that local administration of MV augments antitumour effect of adoptive CD8^+^NKG2D^+^ cells in hepatic carcinoma. They also clarified the mechanisms underlying the increased innate immune stimulation and the possible improvement of viro-immunotherapy [34].

In a study, Wang et al. employed an oncolytic measles virus encoding interleukin 12 (IL-12) to treat colon cancer in vivo and ex vivo to evaluate its actions on the survival and apoptosis of colon cancer cells. A rat model was created to study the immunostimulatory activities and therapeutic effectiveness of vectors encoding an IL-12 fusion protein (MeVac FmIL-12 vectors). MeVac FmIL-12 increased the therapeutic efficiency of tumor therapy [35].

Human cytomegalovirus (HCMV) is a ubiquitous opportunistic species-specific herpesvirus that infects a great part of the population worldwide. Even though HCMV infection often provokes an asymptomatic infection in healthy subjects, it causes significant mortality and morbidity in immunosuppressed subjects [36]. Preliminary findings sustain the employ of CMV in viral oncolytic treatment a viable option [37].

Coxsackievirus is a component of the family Picornaviridae, genus Enterovirus. Coxsackievirus, has entered into clinical trials and their effectiveness and security have been reported with minor side effects. Finding reported from numerous clinical trials in subjects with tumors have been reported in detail. Some preclinical experimentations of these oncolytic viruses have confirmed encouraging results, principally when administered in combination with chemotherapeutic drugs [38].

Newcastle disease virus (NDV) is an RNA virus belonging to the Paramyxoviridae family. In nature, NDV mainly infects animals, but causes no threat to human health. Preclinical and clinical reports have established that NDV has oncolytic capacities and can powerfully activate antitumor immune responses. NDV selectively infects, and lyses tumor cells by exploiting defective antiviral defenses in cancer cells. Inflammation within the tumor microenvironment in response to NDV leads to the recruitment of innate and adaptive immune effector cells, presentation of tumor antigens, and induction of immune checkpoints [39].

Finally, mumps virus is an enveloped, non-segmented, negative-sense, single stranded RNA virus that has a spherical or a pleiomorphic shape of ~200 nm. Mumps virus is responsible for an acute viral infection, spread by respiratory droplets [40]. Recently, recombinant Mumps virus was shown to have oncolytic activity and may act a cancer therapeutic agent [41].

## 2. Antitumoral Action of Oncolytic Viruses

**Figure 1:**
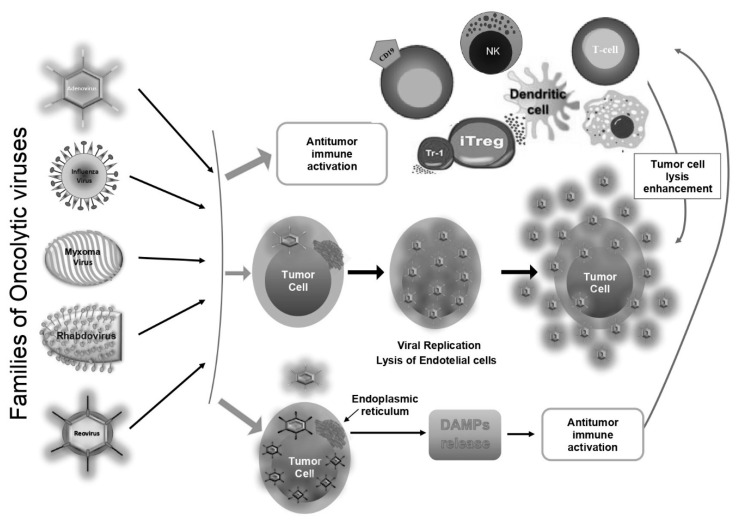
Some mechanisms of action of Oncolytic viruses.

Previously, it was hypothesised that OVs’ antineoplastic action was mainly owed to their ability to contaminate, proliferate and destroy tumour cells and the immediate destructive effect was believed to be the unique action of OVs. It is now recognized that OVs operate via a multiplicity of effects, including mutation of the tumour milieu and a composite change of the action of immune effectors (Figure 1). OVs redesigns the pro-tumour milieu to an inflammatory and antitumor milieu by affecting tumour correlated cells such as fibroblasts, macrophages, endothelial cells, and neutrophils [42].

Discharged tumoral antigens activate non-infected neoplastic cells’ immune lysis, boosting its curative action. Moreover, it has been reported that OVs stimulates peril indicators and immune-mediated cell death mechanisms, activating the activity of immune effectors versus tumour cells [43]. For this reason, OVs are recognized as immunotherapeutic factors.

However, OVs’ mechanism of action is more complex, and a central function is carried out by oxidative stress, the importance of which in the genesis of treatment of oncological diseases in general and of onco-haematological pathologies in particular is well known [44,45,46,47,48,49].

Influenza A virus cause the production of radical oxidative species (ROS). Cells detect ROS via Kelch-like ECH-associated protein 1 (KEAP1) which provokes an effect reliant on dosage. At small percentages of ROS the transcription factor nuclear factor erythroid 2-related factor 2 (NRF2) is stimulated by KEAP1 and supports cell survival. Moreover, KEAP1 connects to the mitochondrial phosphatase PGAM5 and deactivates it. At higher percentages, KEAP1 disconnects from the phosphatase, causing the initiation of oxeiptosis, a type of programmed cell death independent from caspases [50].

Furthermore, the contact of cells to viruses generates structural modifications of the endoplasmic reticulum (ER), causing the liberation of molecules called damage associate molecular pattern (DAMPs) and starting a protective immunologic activity against the tumour [51]. DAMPs involve and stimulate immune cells with presentation of new antigens to T lymphocytes to eradicate tumours. Antigen presenting cells (APCs), such as dendritic cells (DCs), stimulate the delivery of α and β IFNs modulating macrophages to a different phenotype (M1-phenotype) and stimulating the production of IL-1 α and tumour necrosis factor-α (TNF-α). Factors discharged by stimulated DCs contribute to the stimulation of natural killer (NK) cells to eliminate tumour elements via necroptosis [52,53,54]. A recent article involves necroptosis signalling through Receptor Interacting Protein Kinase 3 (RIPK3) in provoking powerful anti-tumour immune responses separate from the phenotype provoked by DAMP discharge and mixed lineage kinase domain-like pseudokinase release [55]. Influenza A virus presence can be detected by the host via Z-DNA binding protein 1(ZBP1). This can cause cellular death through necroptosis or programmed cell death [56]. Contrariwise, the cellular inhibitor of apoptosis protein 2 (cIAP2) has been demonstrated to defend against necroptosis caused by influenza A virus [57].

Other possible mechanisms include the regulation of proliferation by transcriptional aiming or substitution of viral promoters with tumour promoters [58].

In any case, genetic alteration and the resulting decrease in toxicity and the increase in effectiveness is closely correlated to the type of virus used. For instance, to enhance the therapeutic efficacy of conditionally proliferating Adenoviruses (CRAds), two main techniques are presently being used. It is possible to create chimeric vectors, where the entire fibre or the knob portion is replaced with that of a different type of Ad, causing a reduced liver damage ascribed to minor hepatic tropism, and augmented infection of the cancer cells by coxsackie adenovirus receptor (CAR)-independent transduction [59,60,61].

A diverse approach is based on the addition of beneficial genes into the genetic material of a changed CRAd. This method can not only immediately destroy tumour cells by lysis but also increase the number of therapeutic genes, provoking a longer transgene expression within tumours [62,63]. Then, the incorporation of therapeutic genes into the viral genome is used to create “reinforced” OVs, which have genes with anti-angiogenic properties, as well as the ability to produce cytokines provoking an immune activity against tumour cells [64]. For instance, this result can be attained by transforming a virus with a proapoptotic factor such as interleukin-24 (IL-24). It was reported that this cytokine makes tumour cells sensitive to programmed cell death after an infection due to influenza A virus [65]. However, IL-24′s apoptotic action is based on a second signal, specifically the stimulation of the TLR3 by viral RNA. However, the in vitro effects of an influenza virus armed with this cytokine might be problematic to transmute to clinical evaluations, as IL-24 inhibits viral proliferation [66].

Finally, it is possible to act on OVs to generate suicide genes which ease the transformation of inoperative prodrugs into active elements [67].

Studies on oncolytic viruses allowed the Food and Drug Administration and European Medicines Agency authorization for the clinical employ of an OV, Talimogene laherparepvec (T-VEC) [68]. Currently, several OVs have been authorised for tumour therapy. An ECHO virus type 7 (ECHO-7) for the therapy of melanoma, an engineered HSV-1 presenting GM-CSF, for metastatic melanoma, and H101 a mitigated adenovirus for the therapy of head and neck carcinoma [58,69,70].

The aim of our work is to evaluate the results present in the literature on the use of OVs in the therapy of haematological neoplastic pathologies such as multiple myeloma, acute and chronic myeloid leukaemia, and lymphoproliferative diseases (Table 1).

### 2.1. Oncolytic Viruses and Haematological Malignancies

#### Oncolytic Viruses and Multiple Myeloma

Despite the progress made in the field of GM-CSF treatment [71,72,73,74], the disease remains an uncurable pathology which requires new types of approach.

MM is an essentially immunosuppressive neoplastic disease, which forces the tumour milieu to threaten tumour-associated dendritic cells and macrophages to a pro-tumoral, immune-suppressive setting [75]. Immune reactions are further reduced in MM subjects by the same drug treatment, particularly with corticosteroids, alkylators, and proteasome inhibitors [76].

In contrast to traditional methods of tumour vaccination, virotherapy supports a self-tuning incitement to the immune response against MM cells. After viro-treatment, virus-caused tumour cell destroying endures till immune reactions have augmented appropriately to eradicate the infection.

Numerous investigations have evaluated the possibility to use measles virus (MV), an RNA virus of the Paramyxoviridae family, as a treatment for MM [77]. MVNIS is a recombinant MV (Edmonston strain) modified to present the human thyroidal sodium iodide symporter (NIS) to ease imagining of virus infected cells in vivo using single-photon emission computed tomography. This modified virus uses CD46 receptors to pass in cells and to cause intercellular union of infected cells with uncontaminated cells, causing the creation of not vital multinucleated syncytia [78]. Myeloma cells present an increased expression of CD46 and are consequently extremely vulnerable to MVNIS destroying [78]. Recently, a phase 1 experimentation assessing the security and evaluating maximal tolerated dose (MTD) of intravenous (i.v.) dispensation of MV-NIS in subjects with MM relapsed or refractory (RRMM) was performed [79]. RRMM subjects received the treatment as a monotherapy without having severe toxicities, and the MTD was not achieved. One RRMM subject obtained a complete remission (CR), while a smaller reduction in myeloma-specific IgG was reported in other subjects. Afterward, other RRMM subjects were included in an extension cohort [80].

As MM plasma cells lines and primary MM plasma cells present the carcinoma-selective protein DF3/MUC1 and some integrins are needed for the entrance of the viruses into the cells; it was reasonable to use Ads that transport genes under the influence of DF3 promoter. Different experimentations reported the effectiveness of Adenovirus to transport the thymidine kinase (TK) gene into MM plasma cells like RPMI 8226 and OCI-My5, and into primary patient plasma cells [81]. It was established that transduction of Ad carrying the TK gene under the influence of the DF3 promoter (Ad.DF3-NK) and the administration of ganciclovir, an anti-viral drug removed an enormous amount of infected RPMI 8226 and OCI-My5cells. Notably, not tumoral human hematopoietic cells were not touched by this therapeutical approach [82]. In a different experimental model performed employing a CRAd containing a CD40 ligand transgene (AdEHCD40L), it was demonstrated an increased block of MM cells proliferation [83], and an AdEHCD40L provoked programmed cell death was highlighted in MM cells. Experimentations carried out by the same group employing a severe combined immunodeficiency disease (SCID) mouse model pre-implanted with RPMI 8226 and treated with AdEHCD40L, demonstrated a 50% reduction in MM cells in comparison to controls (28% MM cells decrease) [83].

Notwithstanding the encouraging results obtained employing Ad as a possible treatment against MM cells, the main problem for its use in clinical practice is its great immunogenicity with the stimulation of high concentrations of anti-Ad sero-reactivity in human populations [84]. For this reason, other viruses have been tested for their anti-myeloma activity.

Several experimentations performed in vitro, in vivo, and ex vivo have demonstrated that reovirus displays lytic effects against MM cells [85]. Remarkably, investigations have reported that this virus does not damage normal hematopoietic stem cells. Numerous analyses have assessed reovirus’ ability to eradicate infected MM plasma cells [86,87].

It is important to point out that the combined treatment OVs-chemotherapeutics could increase therapy effectiveness and also reduce the appearance of treatment-resistant escaping variants.

For instance, myeloma plasma cells are extremely susceptible to VSV’s lytic actions and are responsive to bortezomib’s cytotoxic actions Nevertheless, Bortezomib blocked VSV-caused NF-κB stimulation, which is essential to spread efficiently. In MM experimental models in vivo, the combined treatment considerably decreased tumour load in comparison to single elements [88].

Furthermore, several vaccination methodologies are being assessed in experimentations to increase the immune response to tumour-associated antigens (TAAs) [89], and oncolytic viruses are also being assessed [90]. OVs offer a platform to pursue TAAs increasing the cross presentation of discharged cellular proteins by cancer-resident APCs.

This result was validated by several experimentations. Packiriswamy et al. evaluated T-cell activities against a group of MM TAAs employing PBMC derived from MM subjects before and after utilization of a MV-NIS [91]. MV-NIS treatment remarkably increased T-cell activities against MAGE-C1 and MAGE-A3. Remarkably, one MM subject who obtained CR after MV-NIS treatment had intense T-cell reactions to MS proteins and to most of the examined TAAs. These findings confirmed that OVs can operate as an antigen vaccine, rising the activity of T-lymphocyte against TAAs in MM subjects [91].

### 2.2. Oncolytic Viruses and Acute Myeloid Leukemia

More than a third of subjects with acute myeloid leukemia (AML) may be resistant to treatment, and a greater part of subjects who obtain a CR will undergo relapse. These AML patients present a disease resistant to chemotherapy for which novel therapeutical approaches are indispensably necessitated.

Oncolytic virotherapy is attractive in a pathology such as AML, where the proliferating leukemic elements are distributed intravascularly and in different tissues. The capability of OVs to continuously contaminate tumour cells also contains the capacity for eliminating minimal residual disease [92].

In an experimental model, AML cells were infected with measles vaccine virus either presenting green fluorescent protein (GFP) or reinforced with super cytosine deaminase, which transforms the prodrug, 5-fluorocytosine, into 5-fluorouracil [93]. Findings determined that measles vaccine virus contaminated the leukemic cells and decreased the amount and survival of leukemic blasts through the stimulation of programmed cell death. The transformation of 5-fluorocytosine to 5-fluorouracil exercised a powerful additional action against tumour cells. This method generates the functional drug in specific sites and may overwhelm several limits and allows to use 5-fluorouracil in the therapy of AML [93].

Unlike the traditional chemotherapies, virotherapy does not influence non-malignant cells, especially those of the bone marrow (BM), and allows formation of normal blood components to be maintained after the therapy. Furthermore, while the direct destruction of leukemic cells was less marked in the ex vivo set, in comparison to AML cell lines, the metabolic function of primary cells was intensely altered [94].

Reovirus is an OV that has demonstrated preclinical effectiveness in the therapy of several tumours and has been verified in phase III trials. In a report, researchers evaluated reovirus’ direct action on AML. Reovirus was demonstrated to proliferate in AML cell lines and to destroy them, and to decrease cell vitality in primary AML cells [95].

As far as the mechanisms by which reoviruses carry out their action are concerned, they provoke an activation of NK cells with augmented NK degranulation and IFN delivery. Stimulation of NK cells is due to a stimulation of DCs and successive relation with NK cells, as lonely NK cells are not stimulated by the virus [96,97].

A different oncolytic virus used for AML therapy is MYXV, a member of the Poxviridae family. Notably, there are no described anti-MYXV antibodies in humans. Madlambayan et al. assessed how MYXV affects AML cells. MYXV inhibited myeloid sarcoma development and bone marrow engraftment of two human AML cell lines [98]. The decrease in engraftment after ex vivo MYXV therapy was dependent from the dosage and necessitated a minimum multiplicity of infection (MOI) of 3.

Moreover, MYXV presents activity against AML tumor xenografts, and is able to aim leukaemia cells while saving normal hematopoietic stem cells [99,100].

A direct pro-apoptotic action of Cytomegalovirus (CMV) on AML cell lines has also been demonstrated [101]. This anti-leukemic action is due to an effect caspase-dependent and could justify the reduced relapse percentages in AML subjects with CMV recurrence [101].

The Kuykendall strain of coxsackievirus A21 (CVA21), is an enterovirus that uses Decay Accelerating Factor (DAF) to connect to cells and Intercellular Adhesion Molecule 1 (ICAM-1) for entrance. An increased expression of ICAM-1, reported on numerous neoplastic cells, can be used as a prognosticator of tumour cell responsiveness to CVA21-caused lysis [102,103,104]. CVA21 provoked a powerful immune response against tumour via several mechanisms such as a cytokine-provoked bystander death, an increased NK -mediated cytotoxicity through the action of tumour-specific T lymphocytes. Relevantly, immune-mediated death of AML cells was detected, notwithstanding AML cells being resilient to CVA21 lysis. Furthermore, the role of IFN for NK cell stimulation was established, and it was determined that ICAM-1 and plasmacytoid DCs were essential modulators of this phenomenon [105].

If good results in oncolytic AML therapy have been achieved with the OVs mentioned so far, adenoviruses are undoubtedly among the most commonly used oncolytic viruses. SG235-TRAIL, an adenovirus holding an Ad5/F35 chimeric fibre and engineered with an antitumor gene TRAIL, operated with homoharringtonine in leukemia cell lines with a synergistic effect [106].

Lately, Wang et al. built the adenovirus rAd5pz-zTRAIL-RFP-SΔ24E1a (A4), which has the capsid protein IX connected to TNF-related apoptosis-inducing ligand (TRAIL) and causes a higher infection of tumour cells and an enhanced tumour aiming [107]. To increase the beneficial activity of A4, they produced a different form of A4, zA4, by covering A4 with further TRAIL that is combined with a leucine zipper-like dimerization domain (ZA4). ZA4 provoked a significant block of the growth of AML cells that showed adequate concentrations of TRAIL-related receptors. ZA4 also caused an increased anti-AML action in vivo in comparison to A4. Moreover, they demonstrated that the ginsenoside Rh2 increased the presence of TRAIL receptors and therefore increased the antineoplastic action of zA4 [107].

Rhabdoviruses (RVs), such as VSV and Maraba virus, are presently being also investigated as antitumor agents. Via cell destruction and stimulation of anticancer immune reaction, RVs are self-intensifying lytic mediators. However, although RVs are being employed to cure several types of tumours, their use in hematopoietic malignancies is made difficult by numerous elements such as reduced virion proliferation and decreased spread between leukemic cells [108]. Batenchuk et al. hypothesised that the obstacles to virotherapy in haematological diseases may be overcome by dispensation of high doses of non-proliferating Rhabdovirus [109]. They have built a technique to generate non-proliferating rhabdovirus-originated particles (NRRPs). The relevance of NRRPs was established in AML subjects with great-load chemo-resistant disease. NRRP treatment induced programmed cell death in myeloblasts from subjects with CML in acute blast crisis. Normal cells from healthy BM were not altered [109]. This finding implies that notwithstanding NRRPs’ powerful tumoricidal action, the reduction of leukocytes generally detected after induction and consolidation chemo-treatment could be prevented by employing NRRP-based protocols, with a relevant reduction in adverse events.

A further opportunity may arise from VSV–interferon β (IFNβ)– NIS, a VSV coding IFNβ and the NIS reporter. Syngeneic AML C1498 cells reacted to treatment with VSV-murine IFNβ (mIFNβ)-NIS according to the dosage employed. Imaging for NIS expression demonstrated strong virus presence within the cells. Infection did not augment programmed death ligand 1 (PD-L1) on leukemic cells. When VSV-mIFNβ-NIS was combined with anti-PD-L1 antibody (Ab) treatment increased antineoplastic activity with respect to therapy with virus alone or Ab alone. Moreover, the combined treatment remarkably increased the survival of animals with no sign of side effects, in comparison to anti-PD-L1, or virus alone. A rise in the number of CD4 and CD8 cells in tumours was reported. Reduction of NK or CD8 cells, but not CD4 cells, provoked a decrease in antileukemic action in the VSV/anti-PD-L1 group [110]. Cells from chronic myelomonocytic leukemia and acute myelomonocytic leukemia seem to be particularly vulnerable to VSV.

The use of rhabdoviruses could also have other implications in AML treatment, such as overcoming a chemoresistance. An increased expression of myeloid cell leukemia 1 protein (Mcl-1), a member of B-cell lymphoma 2 (Bcl-2) family member, participates to treatment resistance. VSV has been recognized as an lytic virus that powerfully disturbs the production of new proteins of infected cells. It was reported that after infection, Mcl-1 protein concentrations quickly dropped. Mcl-1 reduction was an effect of proteasomal degradation. Mcl-1 rescue blocked programmed cell death. A combined treatment with VSV virotherapy and demonstrated a synergic action in comparison to virus alone or chemotherapy alone, which could be regressed by RNA interference of Bax and Bak proteins (pro-apoptotic elements) [111]. Furthermore, in an animal experimental model, combined treatments of doxorubicin and VSV demonstrated an increased efficiency in comparison to VSV or doxorubicin alone [111].

A completely different attempt implicates the employ of oncolytic vaccinia virus (OVV).

Beclin-1 is a protein that has been correlated to tumour suppression. Inhibition of Beclin-1 provokes an increased tumorigenesis [112], while an increased expression of Beclin-1 reduces the development of cancers in animal experimentations [113]. Beclin-1 alteration was reported in several forms of tumour cells. A report has stated that in AML and acute lymphoblastic leukemia, Beclin-1 expression was considerably reduced in comparison to controls and that reduced Beclin-1 gene expression was correlated with minor survival [114].

A novel OVV showing Beclin-1 (OVV-BECN1) was examined for its oncolytic effect in leukemia. Findings demonstrated that the OVV presented greater contagion for leukemic cells. OVV-BECN1 provoked relevant autophagic cell death in wild-type leukaemia cell lines in vitro and in vivo models [115].

In an U937 AML animal model, MV demonstrated higher tumour reduction and extended survival. Moreover, MV destroyed leukemic cells from 16 out of 20 AML subjects and produced more effective action on 11 AML subjects when dispensed with Aracytin-C [116].

Finally, particular conditions can determine a different effectiveness of viruses’ oncolytic activity. Telomeres are the nucleoprotein formations at the extremity of chromosomes, which diminish after every cell division. Several neoplastic cells carry out a telomere elongating system to decrease telomere reduction and so to allow limitless cellular growth [117]. Tumours using the alternative lengthening of telomeres (ALT) system for telomere preservation are often problematic to cure and have a bad outcome. They are also generally lacking for presence of ATRX protein, a repressor of ALT activity, and an element of promyelocytic leukemia nuclear bodies (PML NBs) which are essential for immunity to several viruses. Han et al. demonstrated that an HSV-1 missing Infected cell protein 0 (ICP0), a protein that destroys PML NB elements comprising ATRX, was much more effectual in eradicating ATRX-lacking cells. The ability to be sensitive to mutant HSV-1 related inversely with PML protein concentrations [118]. These results offer a base for predicting, based on PML or ATRX concentrations, which haematological diseases will react to an oncolytic herpesvirus.

Selecting the right virus remains an ongoing challenge to treat leukemia. Moreover, as an immune response is essential in the antileukemia action, how this treatment is carried out in treated leukemic subjects who have been submitted to chemo treatment and may have an exhausted immune system remains to be evaluated.

### 2.3. Oncolytic Viruses, Chronic Myeloid Leukemia, and Chronic Lymphocytic Leukemia

An investigation has assessed if a treatment joining the oncolytic action of an adenoviral vector with the concomitant presence of the gene Beclin-1 presented an improvement for chronic myeloid leukaemia (CML) cells resistant to chemotherapy. In the same work, authors have assessed the synergistic actions of SG511-BECN and doxorubicin (Dox) in CML cells [119]. Oncolytic virus SG511-BECN was generated via inserting Beclin-1 gene into the adenoviral structure. This compound has demonstrated considerably enhanced antitumoral effect on multidrug-resistant CML cell line K562/A02, which was due to the stimulation of autophagic cell killing death. Moreover, Doxorubicin could increase the effect of SG511-BECN by increasing the infectious efficacy of the oncolytic Ad without provoking relevant injury to normal human mononuclear cells [119].

### 2.4. Oncolytic Viruses and Lymphomas

It may appear rather strange to use viruses in the therapy of tumour when approximately 15% of all human tumours may be provoked by viruses [120]. Several studies reported the presence of a correlation between lymphomas and viruses such as HIV, Epstein-Barr virus, human T-cell lymphotropic virus, and human herpes virus 8 [121].

Nevertheless, favourable actions of viruses on lymphoma subjects have been acknowledged for long time. It is presumed that fundamental systems of spontaneous disappearance in Hodgkin’s disease and Burkitt’s lymphoma [122,123] after measles contagion are owed to viral oncolysis and virus-caused immune response against the tumour. Moreover, the capacity of several neurotropic viruses to provoke regression of the RPL-12 chicken lymphoma in the pectoral muscle has been reported in old studies [124,125].

However, these findings have been then validated by recent studies. Numerous types of the Newcastle disease virus (NDV) have been used as oncolytics in experimental models. Sanchez et al. evaluated the actions of NDV-MLS, an attenuated type, on a large B-cell lymphoma cell line (SU-DHL-4) and on healthy PBMC. The virus decreased cell vitality in lymphoma cells with respect to controls. No relevant action on PBMC was demonstrated. Destruction of cells was due to programmed cell death as supported by flow-cytometry [126]. Eaton et al. proved an oncolytic action of NDV in vivo. This effect was due to a direct oncolysis and an immune reaction against an ascites lymphoma developed in C3H animals [127].

Several studies reported the proliferation of parainfluenza virus, MV, VSV, and mumps virus, in Burkitt lymphoma (BL) cells in vitro, causing the destruction of 95% of cells [128].

Specific cytopathic actions caused by attenuated MV-Ed were reported against lymphoma cells by other authors [129]. In addition, a completed suppression of BL cells after contamination with reovirus type 3 was described [130].

Vulnerability of primary lymphoid diseases to reovirus was also described. Moreover, normal PBLs and haematopoietic stem cells originated from not neoplastic BM did not support reovirus proliferation, proposing that this virus targets only neoplastic lymphoid cells. However, the follicular lymphoma seemed usually resistant while chronic lymphocytic leukemia (CLL) samples allowed reovirus proliferation. The reason for diversity in tolerance between lymphoproliferative diseases is uncertain and needs to be further investigated. Regarding CLL, a peculiar genetic alteration, probably in the Ras pathway, may cause the perfect milieu for virus proliferation [131,132,133].

A report clarified the mechanism of oncolysis of reovirus in CLL cells. Virus might act via a direct cytotoxic mechanism or an action on the immune system with a stimulation of patient NK cells through a monocyte-derived interferon-α (IFNα)-dependent system. Moreover, reovirus increases cell antibody-dependent cellular cytotoxicity (ADCC)-mediated eradication of CLL cells when administered in combination with anti-CD20 antibodies. These results offer robust preclinical proof to support the usage of reovirus and anti-CD20 drugs for CLL therapy [134].

Other experimentations have confirmed that CLL cells are vulnerable to the action of OVs [135,136], while Medina et al. demonstrated this effect in CLL cells using attenuated Ads with particular mutations in E1 or E2 region [137].

Nevertheless, other studies established that CLL cells were resilient to a diverse RNA OV, VSV, due to an increased production of Bcl-2. However, block of Bcl-2 made cells vulnerable to VSV lysis [138,139].

Mantle cell lymphoma (MCL) may also be an objective of OV treatment. MV-PNP H bind antiCD20 is a CD20-targeted that regulates proliferation of lymphomas in SCID mice in association with fludarabine phosphate. The proliferation of this virus was assessed in disease bulks and cells from MCL subjects. It was demonstrated that the virus is specific for CD20-expressing cells [140]. Authors also evaluated the efficiency of different protocols of dispensation of the virus in association with cyclophosphamide (CPA) in an MCL model. They demonstrated that CPA dispensation before virus increases lytic effectiveness, probably via immunosuppression. Lastly, three courses of consecutive therapy with virus, CPA and fludarabine treatment caused an absolute disappearance of xenografts, while median survival times have risen from 22 to 77 days [140].

Cutaneous T-cell lymphomas (CTCLs) are frequent extranodal lymphomas and are due to a clonal growth of tumoral T-lymphocytes. Even though subjects with patch/plaque disease of less than 10% of the body surface have a regular life expectation, subjects who experience conversion to large-cell lymphoma have a bad outcome with a survival of 2–19 months [141].

In a study, MV vaccine provokes tumour reduction [142]. The action on cell growth was evident in cells establishing aggregates, suggesting a cell-to-cell diffusion of MV and cytolysis due to virus infection. Intra-tumoral (i.t.) administration of rMV, presenting increased GFP provoked total regression of CTCL in mice, while lymphomas with control treatment developed progressively. These findings display the possibility to use MV as a therapeutic approach against CTCL.

Alphaviruses are positive-sense, single-stranded RNA arboviruses that are able of provoking grave disease. The transmission cycle of these viruses is between a mosquito vector and a mammalian host, generally rodents or birds, although epizootic spillover events can happen that cause an infection of humans. Alphaviruses are generally classified as either arthritogenic or encephalitic based on disease symptomology [143].

Sindbis virus (SV) is an alphavirus and SV vectors in combination with α4-1BB monoclonal antibody entirely eradicated a B-cell lymphoma in a preclinical animal model, a result that could not be attained with either treatment alone. Tumor elimination implicates a synergistic action of the combination that significantly boosts T cell proliferation and cytotoxicity, and IFNγ production. Moreover, all animals that survived after therapy acquired long lasting antitumor immunity [144].

Finally, as far influenza virus, it inhibited tumor growth after administration to tumor zone in a dosage of 7-8 lg EID50, as was demonstrated for two different mouse tumor cell strains, Ehrlich’s carcinoma and L-1210 lymphoma. Influenza virus types were different by their antitumor effect which correlated with their interferonogenic action [145].

Moreover, using influenza A matrix protein 1 (MP1), Laurence et al. demonstrated that ex vivo–expanded CD4+ and CD8+ T-APCs presenting a hygromycin phosphotransferase MP1 fusion protein (HyMP1) processed and presented MP1 to autologous human leukocyte antigen (HLA)–restricted, MP1-specific CD4+ and CD8+ cytotoxic T lymphocytes (CTL) precursors. The MP1- specific CTLs are amenable to subsequent genetic modification to express a CD19- specific CAR, designated CD19R, and acquire HLA-unrestricted reactivity toward CD19+ lymphoma tumor targets while maintaining HLA-restricted MP1 specificity. The restimulation of MP1CD19 dual-specific CTLs in vivo by the adoptive transfer of irradiated HyMP1+ T-APCs resulted in the enhanced antilymphoma activity of bispecific effector cells [146].

### 2.5. Oncolytic Viruses in Transplantation and Graft vs. Host

Haematologic stem cell rescue after high dose cytotoxic treatment is widely utilized for the therapy of several hematologic tumours. Gene marking analysis propose that hidden neoplastic cells within the autograft may participate to the recurrence of the disease. Removing of autografts polluted with tumour cells has been ineffective. The specific oncolytic ability of reovirus against several types of tumours in vitro, in vivo, and ex vivo experimental models has been beforehand verified. MYXV particularly eradicated contaminating AML subjects-originated tumour stem cells in explants by ex vivo viral purging prior to re-engraftment [99].

But treatment with oncolytic viruses also seems promising in the case of transplant procedures other than those related to AML. In a study, authors have reported that reovirus can efficiently eliminate tumour cells within autografts. Survival of the cell lines or purified ex vivo tumour cells of CLL, Waldenstrom macroglobulinemia and diffuse large B-cell lymphoma was drastically decreased after reovirus administration. Contrariwise, reovirus eradication of MM, BL, and follicular lymphoma cells was inadequate [137].

Finally, Kim et al. stated the effectiveness of MYXV eradication of tumour cells as a preventive treatment for inhibiting post-transplant EBV-transformed human lymphomas [147].

Oncovirus therapy could favourably take action at other times of transplantation therapy. In spite of the relevant progress made in the last years to decrease the gravity graft-versus-host disease (GvHD) after allo-HSCT, it is the main reason for nonrelapsing tumour mortality, being lethal for up to 20% of patients [148].

Recently, numerous findings have proposed that virotherapy of HSCT samples with MYXV can reduce the negative effects of GvHD, while retaining the favourable GvT actions in the setting of allo-transplantation against MM. In vivo experimentations employing a xenograft model displayed that BM cells treated ex vivo with MYXV and subsequently transplanted into NSG animals significantly decreased mortality in comparison to BM samples that had not been deal with the virus [149]. In vivo experimentations established that ex vivo MYXV therapy decreased the gravity of post-transplant GvHD by taking action on the capability of the donor human T cells to cause aGvHD [150,151].

## 3. Oncolytic Viruses: Possible Combination Therapies

Set on a computational basis, Wodarz states that even if oncolytic virus treatment flops to eliminate a tumour, it can have the possibility to eliminate the sub-group of drug-resistant tumour cells [152]. The justification for this hypothesis originates from a population dynamics theory that is named ‘apparent competition’. Even if two groups do not exactly contend with each other, the fittest group can force the least fit group dead if they are infected by the same pathogen. For instance, if the proliferation percentage of the drug-sensitive tumour cells is higher than that of the resistant tumour cells, then the resistant cells will be dead if a virus can infect both cell groups.

OVs is an encouraging treatment for a broad group of neoplastic diseases but keeps on being an understudied therapy option for haematological malignancies, although tumour virotherapy offers a new approach to treat tumours as agents that do not have cross-resistance with presently available therapies [153,154].

However, only a small part of patients was completely responsive to OV monotherapy. Improved prognosis was achieved using OVs together with other therapeutical approaches, such as immune treatment or in combination with chemotherapy [155,156], indicating that the complete capacity of OVs can be released in association with other therapies.

In any case it is certain that to amplify OV efficacy, the assessment of new associations should be highlighted. An approach could be combining oncolytic CMV treatment with epigenetic treatment [157]. In defence of such assumption, histone deacetylase (HDAC) inhibitors arose as enhancers of virotherapy, producing the concept of “epi-virotherapeutic therapy” [158,159].

A combined treatment with other HDACi, such as valproic acid (VPA), could be assessed to increase the presence of NKG2D ligands on leukemic cells [160] and improve the cytotoxic action of NK cells after OV treatment, or synergize with OVs to increase viral proliferation and cellular lysis [161]. Moreover, hypomethylating drugs, such as decitabine, can augment the presence of TAAs [162]. Consequently, association of viruses such as CVA21 with diverse epigenetic drugs may be useful to stimulate anti-tumour immune responses.

A different approach might be the association of OVs with new oncolytic immune-treatments of tumour, such as the immune checkpoint inhibitors aiming cytotoxic T lymphocyte antigen 4 (CTLA-4) and the PD-1/PD-L1 pathway [163].

It is well known that OVs not only directly destroy tumour cells, but also modify the immune response and the tumour milieu. Several reports have demonstrated that OVs can transform immune cold tumour that were earlier unresponsive to PD-1/PD-L1 inhibitors into immune hot tumour [164]. So, tumours are more vulnerable to immunotherapy, and PD-1/PD-L1 inhibitors may cause actions [165]. The association of the two treatments remarkably increases the amount of tumour-specific CD4+ and CD8+ T cells and increases the immune reaction against the tumour [166]. This association seems to be extremely beneficial in several preclinical models [167,168,169,170].

Finally, treatment with oncolytic viruses could also find a place in what appears to be the latest frontier in the treatment of haematological diseases, the immunotherapy with Chimeric Antigen Receptor (CAR) T Cells.

CAR-T cells are built to code receptors for certain tumour antigens and operate via an MHC-dependent system [171]. However, this treatment has had partial success for several conditions, including the immunosuppressive tumour milieu. One method of overcoming these difficulties of CAR-T treatment is to use CAR-T cells in association with oncolytic viruses as part of a combined procedure [172].

In an NOD/SCID/Il2rg null (NSG) xenograft animal model employing Human pancreas adenocarcinoma ascites metastasis (AsPC-1) cells, Authors demonstrated that cancers treated with the combination of the virus expressing TNF-α and IL-2 (Ad5/3-E2F-d24-TNF-α-IRES-IL-2 [OAd-TNFα-IL2]) along with CAR-T cells resulted in considerably greater accumulation of CAR-T cells at the tumor site, as well as augmented cancer regression [173]. In a different approach, Watanabe et al. increased the effectiveness of CAR-T cell treatment using a combinatorial adenovirus vector (oncolytic adenovirus (Ad5Δ24) and helper-dependent adenovirus expressing a mini anti-PD-L1 antibody (HDAdPD-L1) collectively called CAd-VEC*PDL1*) in conjunction with human epidermal growth factor receptor 2 (HER2)-specific CAR-T cells [174]. Employing this combination in an NSG animal model of prostate cancer xenograft they established that in the presence of HER2-CAR-T cells, the CAd-VEC*PDL1* virus’s expression of anti-PD-L1 antibody at the cancer site was considerably more efficacious at reducing cancer size [174].

## 4. Limitations of Oncolytic Virus Therapy

While the possibility of using oncolytic viruses for tumour immunotherapy is attractive, it also has its inadequacies. As we have seen above, most OVs show modest antitumour results as a monotherapy. This is probably due to neutralizing antibodies [175], and high incidence of pre-existing neutralizing antibody against specific viruses is the biggest limitation for this treatment [176].

To elude pre-existing immunity, different approaches have been practiced, such as change of capsid protein, using alternative serotypes, or physically protecting virus particles [177,178]. A new method using molecular redirecting of anti-Ad antibodies to tumour cells demonstrated pre-existing antivirus antibodies can also be employed as powerful antitumor instruments [179]. This approach involves the use of a recombinant bifunctional adapter protein with the ability to catch anti-Ad antibodies but also identifies tumour cells via a polysialic acid-specific single-chain variable fragment [180].

A different attempt to neutralize antibodies involves the use of viruses that are infrequent or lacking in the population such as rare serotype Ad and Newcastle disease virus. However, this approach could cause new biological risks to possible virus adaptation to humans [181,182].

Regardless of these considerations, it is in any case difficult to convert the results achieved in in vitro experimentations into in vivo clinical protocols. Tumours such as lymphomas are three-dimensional formations, contrasting to two-dimensional cell cultures. To integrate the three-dimensional aspect of lymphomas in an experimental system, spheroid culture models can be employed. It appears easier for a virus to contact all tumour cells in a monolayer than in the three-dimensional formation of real tumours. Moreover, spheroids produce great quantity of cathepsins (as several tumours do), and these substances can extracellularly transform the virus particles into a layout that can contaminate cells regardless of canonical receptors. This system imitates the proteolytic stimulation of the virus particles in endosomes.

However, it has been beforehand discovered that certain cancer cells avoid oncolytic infection in monolayer, while the cells are infected as spheroids [183].

**Table 1:** Main in vitro and in vivo studies in Hematologic Malignancies.

Study	Disease	Virus	Possible Disadvantages	Ref.
**In vitro**	Multiple Myeloma cell line	Measles virus		[78]
	Multiple myeloma and breast cancer cells	Adenovirus		[81,82,83]
	Multiple myeloma cell lines	Reovirus		[85]
**In vivo**	Multiple Myeloma	Adenovirus	Induction of proinflammatory cytokines. Neutralization by serum factors Sequestration in liver and spleen	[83,182]
	Multiple myeloma	Reovirus	Unknown	[85]
	Multiple myeloma	Measles virus	Increased unwanted pathology	[80,182]
**In vitro**	Acute Myeloid Leukemia	Measles virus		[93]
	Acute myeloid leukemia	Reovirus		[95]
	Acute myeloid leukemia, FLT3 mutant acute myeloid leukemia cells	Myxoma virus		[98,99,100]
	Kasumi-1 (AML), SD-1 (BCR-ABL-positive ALL)	Cytomegalovirus		[101]
	Acute myeloid leukemia cells	Coxsackievirus		[105]
	Kasumi-1, KG-1, HL-60, U937 AML cell lines	Adenovirus		[106,107]
	High-burden multidrug-resistant AML cells A549, HEPG2, Huh-7 cell lines	Non-replicating rhabdovirus-derived particles, Vesicular Stomatitis Virus		[109,111]
	Wild-type leukemia cells, Multiple myeloma cell lines	Vaccinia virus		[115]
	Baby hamster kidney-21 cells	Herpes Simplex Virus-1		[118]
**In vivo**	Acute Myeloid Leukemia	Adenovirus	Sequestration in liver and spleen	[107]
	Acute myeloid leukemia	Vesicular Stomatitis Virus	Neurotoxicity	[111,182]
	Acute myeloid leukemia cells	Measles virus	Increased unwanted pathology	[116]
**In vitro**	Chronic Myeloid Leukemia cells	Adenovirus		[119]
	Human and canine lymphomas	Newcastle disease virus		[126]
	Burkitt lymphoma cells, Cutaneous T-cell lymphoma	Measles virus		[128,142]
	Burkitt’s tumor cells, Chronic lymphocytic leukemia	Reovirus type 3		[130,134]
	Chronic lymphocytic leukemia	Adenovirus		[137]
**In vivo**	Mantle cell lymphoma, Cutaneous T-cell lymphoma	Measles virus	Increased unwanted pathology	[140,142,182]
	Non Hodgkin lymphoma- A20 lymphoma	Sindbis virus	Unknown	[144]

## 5. Conclusions

Without a comprehensive environmental risk assessment, new treatments will not be admitted by regulatory agencies like EMA and FDA. Particularly with oncolytic agents, the possibility that they might become more virulent or the chance to express transgenes that modify the behaviour of the virus, should be excluded.

Moreover, further experimentations are essential to recognize the most efficient virus or treatment combinations for specific haematological diseases, and the associations able to induce the most vigorous immune response. It is also essential to recognize the best administration schedules. Experimentations have evidently revealed that sequencing and dosages for both OVs and drugs are decisive to achieve an antitumoral action.

In the future, further studies on viruses’ oncolytic activity could possibly turn the threat into a treatment.

## Abbreviations

OVs	Oncolytic viruses
IFNs	Interferons
TLR	Toll-like receptors
Ad	Adenovirus
MYXV	Myxoma virus
VSV	Vesicular Stomatitis Virus
MYC	Myelocytomatosis
eIF2B	Eukaryotic translation initiation factor 2B subunit alpha
Rae1	ribonucleic acid export 1
Nup98	Nucleoporin 98
eIF4E	Eukaryotic translation initiation factor 4E
KEAP1	Kelch-like ECH-associated protein 1
ROS	Reactive oxygen species
NRF2	nuclear factor erythroid 2-related factor 2
PGAM5	Phosphoglycerate mutase 5
ER	Endoplasmic reticulum
DAMPs	Damage associate molecular pattern
APCs	Antigen presenting cells
DCs	Dendritic cells
TNF-α	Tumour necrosis factor-α
IL-1	Interleukin 1
NK	Natural killer
RIPK3	Receptor Interacting Protein Kinase 3
ZBP1	Z-DNA binding protein 1
cIAP2	cellular inhibitor of apoptosis protein 2
CAR	Coxsackie adenovirus receptor
CRAds	Conditionally Replicative Adenoviruses
T-VEC	Talimogene laherparepvec
ECHO	**7** ECHO virus type 7
HSV-1	Herpes simplex virus 1
GM-CSF	Granulocyte-macrophage colony-stimulating factor
MM	multiple myeloma
MV	Measles virus
MVNIS	Measles virus encoding the human thyroidal sodium iodide symporter
MTD	Maximal tolerated dose
RRMM	Multiple myeloma relapsed or refractory
CR	Complete remission
MUC1	Mucin 1
TK	thymidine kinase
AdEHCD40L	CD40 ligand transgene
SCID	Severe combined immunodeficiency disease (SCID)
NF-κB	nuclear factor kappa-light-chain-enhancer of activated B cells
TAAs	Tumour-associated antigens
PBMC	peripheral blood mononuclear cell
MAGE	Melanoma-associated antigen
AML	Acute myeloid leukemia
GFP	Green fluorescent protein
BM	Bone marrow
MOI	Minimum multiplicity of infection
CMV	Cytomegalovirus
CVA21	Coxsackievirus A21
DAF	Decay Accelerating Factor
ICAM-1	Intercellular Adhesion Molecule 1
TRAIL	TNF-related apoptosis-inducing ligand (TRAIL)
A4	rAd5pz-zTRAIL-RFP-SΔ24E1a
ZA4	Zipper-like dimerization domain
RVs	Rhabdoviruses
NRRPs	Non-proliferating rhabdovirus-originated particles
PD-L1	Programmed death ligand 1
Ab	Antibody
Mcl-1	Myeloid cell leukemia 1 protein
Bcl-2	B-cell lymphoma 2
Bax	Bcl-2-associated X protein
Bak	Bcl-2 homologous antagonist killer
OVV	Oncolytic vaccinia virus
ALT	Alternative lengthening of telomeres
PML NBs	Promyelocytic leukemia nuclear bodies
CML	Chronic myeloid leukaemia
NDV	Newcastle disease virus
CLL	Chronic lymphocytic leukemia
ADCC	Antibody-dependent cellular cytotoxicity
MCL	Mantle cell lymphoma
CPA	Cyclophosphamide
CTCLs	Cutaneous T-cell lymphomas
EBV	Epstein-Barr Virus
ICP0	Infected cell protein 0
GvHD	Graft-versus-host disease
allo-HSCT	Allogeneic Hematopoietic Cell Transplantation
HDAC	Histone deacetylase HDAC
VPA	Valproic acid
NKG2D	Natural Killer-gene complex 2D
CTLA-4	Cytotoxic T lymphocyte antigen 4
CAR	Chimeric Antigen Receptor
EMA	European Medicines Agency
FDA	Food and Drug Administration
